# Corneal higher-order aberrations after cataract surgery: Manual phacoemulsification versus femtosecond-laser assisted technique

**DOI:** 10.1177/1120672121990611

**Published:** 2021-01-26

**Authors:** Dominika Pohlmann, Daniel Pilger, Eckart Bertelmann, Christoph von Sonnleithner

**Affiliations:** Charité *–* Universitätsmedizin Berlin, corporate member of Freie Universität Berlin, Humboldt-Universität zu Berlin, and Berlin Institute of Health, Berlin, Germany

**Keywords:** Cataract surgery, higher-order aberration, femtosecond-laser assisted, ray tracing

## Abstract

**Purpose:**

To compare and evaluate corneal higher-order aberrations (c-HOA) between conventional manual phacoemulsification (Phaco), femtosecond laser-assisted cataract surgery (FLACS), and femtosecond laser-assisted cataract surgery with astigmatic keratotomy (FSAK).

**Methods:**

In this retrospective single center study, 53 healthy individuals with cataract (73 eyes) underwent phacoemulsification with implantation of an intraocular lens. Three groups were formed: group A, Phaco (*n* = 27 eyes of 21 patients); group B, FLACS (*n* = 25 eyes of 15 patients); group C, FSAK (*n* = 21 eyes of 17 patients). An iTrace aberrometer (Tracey Technologies, Houston, TX, USA) was used to perform aberrometry with a pupil scan size of 5.0 mm. We used ANOVA analysis and the paired sample *t*-test for statistical analysis.

**Results:**

There was no difference in total c-HOA between the groups prior to surgery (*F*(2,66) = 2.2, *p* = 0.128), but some evidence for a difference between the groups after surgery (F(2,65) = 3.87, *p* = 0.025). After surgery, total c-HOA increased in all groups, but the greatest increase occurred FSAK.

**Conclusion:**

Manual phacoemulsification and femtosecond laser-assisted cataract surgery seem to have less impact on corneal higher-order aberrations than the combination of femtosecond laser-assisted cataract surgery with astigmatic keratotomy.

## Introduction

Cataract surgery is considered as one of the safest and most efficient surgical procedures in ophthalmology. Over the past decades, innovations in cataract surgery have increased accuracy and improved refractive outcome. The most common technique nowadays is manual phacoemulsification (Phaco). On the other hand, femtosecond laser-assisted cataract surgery (FLACS) is becoming more popular.

The three main steps performed by FLACS are: corneal incisions, capsulotomy, and lens fragmentation. In addition, it is possible to reduce regular astigmatism with keratotomies (femtosecond laser-assisted cataract surgery with astigmatic keratotomy, FSAK).^1^ The procedure is safe and effective.^2^ There are many proven advantages over manual surgery, such as better incision quality with reduction of induced astigmatism, increased reliability, reproducibility of the capsulotomy with increased stability of the implanted lens, and reduced use of ultrasound.^2,3^ Due to its precision, it may also be gentler to the internal structures of the eye.^4^ However, recent studies have reported that there is no superiority to manual Phaco in terms of primary outcomes, such as visual and refractive outcomes.^4,5^

The visual quality of the eye depends on several optical factors, the most important one being lower-order aberrations. In addition, corneal higher-order aberrations (c-HOA) can influence visual quality.^6^ While the lower-order aberrations consist of hyperopia and myopia (defocus), as well as astigmatism, the c-HOAs compromise many varieties of aberrations such as coma, spherical aberration, and trefoil.^6^ Although conventional spherical intraocular lenses (IOL) provide good visual acuity, the spherical aberration is increased and simultaneously the visual quality worsens.^7^ Therefore, aspheric IOLs (AIOL) are used to reduce c-HOAs and improve contrast sensitivity and image quality.^8–13^ Also, the size of corneal incision might have an impact on c-HOAs in cataract surgery.^14^ Little is known about the effect of FLACS on c-HOAs.

In this study, we compare and evaluate c-HOAs between conventional manual Phaco, FLACS, and FSAK.

## Patients and methods

Between April 2017 and December 2017, 53 patients (73 eyes) were enrolled at the Department of Ophthalmology, University Medicine Charité, Berlin, Germany. Inclusion criteria were patients with uncomplicated cataract requiring surgery, a minimum age of 18 years and preoperative, regular corneal astigmatism (less than 0.75 diopters in group A and B; more than 0.75 diopters in group C). Exclusion criteria were any clinically significant corneal abnormalities including endothelial dystrophy, superficial punctate keratitis, poorly dilated pupil, or other significant ocular abnormalities. Additionally, no cases had prior corneal surgery (e.g. laser-vision correction). This study was performed in accordance with the Declaration of Helsinki.

The femtosecond laser procedures were performed by one experienced surgeon (C.v.S). All the cataract surgeries (including the patients prior treated with femtosecond laser) were performed by another experienced surgeon (E.B.).

All patients underwent a full ophthalmic assessment by the same physician (D.P.) pre- and postoperatively after 4 weeks, including slit lamp examination and fundoscopy. Intraocular eye pressure was measured with Goldmann applanation tonometry. On study days, all patients’ demographics were documented. This data included best corrected visual acuity (BCVA). Optical biometry was performed using the IOL-Master 700 (Carl Zeiss Meditec AG; Jena, Germany) to measure keratometry and axial length. For IOL calculation, SRK/T- and Haigis-formula were used, depending on the length of the patients’ eyes. Aberrometry with a pupil scan size of 5.0 mm was performed using iTrace aberrometer (Tracey Technologies, Houston, TX, USA).

### Surgical techniques

Three groups were formed based on the surgical technique and the preoperative corneal astigmatism.

In groups A and B, we enrolled patients with regular corneal astigmatism of less than 0.75 diopters (D). In group C we enrolled patients with regular corneal astigmatism of more than 0.75 D.

Preoperatively, we used tropicamide/phenylephrine eyedrops for pupil dilation in all eyes.

In group A (Phaco) cataract surgery was undertaken in peribulbar anesthesia (Xylocaine® 2%, Naropin®, Hylase “Dessau”®). Oculopression was applied with approximately 25 mmHg for 10 min directly before each operation. The 2.2 mm incision was placed in dependence of the steep meridian of the cornea (superior or temporal). Additionally, we made two 1 mm incisions. The continuous curvilinear capsulorrhexis, hydrodissection, hydrodelineation, phacoemulsification, and aspiration of cortical material were performed under protection with Amvisc®. To perform Phaco we used the Centurion® Vision System (Alcon, Fort Worth, TX, USA).

In group B (FLACS) corneal incisions, capsulotomy, and lens fragmentation were performed with the LenSx® (Alcon, Fort Worth, TX, USA) in topical anesthesia (oxybuprocaine eye drops and lidocaine gel). The main corneal incision was always placed in the superior position. After the femtosecond laser-assisted procedure, the cataract surgery was concluded using the Centurion® Vision System (Alcon, Fort Worth, TX, USA).

In group C (FSAK) femtosecond laser procedure was performed in the same way as in group B. In addition, we used the femtosecond laser for astigmatic keratotomy to reduce regular corneal astigmatism using Donnenfeld Nomogram. One or two keratotomies were used depending on the axis of the astigmatism. The depth was 80% of the corneal thickness.

In all groups we implanted randomly different IOLs (Tecnis ZCB00, Abbott Medical Optics, Santa Ana, CA, USA; CT Asphina 409M, Carl Zeiss Meditec, Jena, Germany; Hoya iSert 251, Hoya, Tokyo, Japan).

### Statistical analysis

All data was entered into a database and checked for data entry errors. Differences in c-HOAs between the groups were compared pre and post-surgery using an ANOVA analysis. We evaluated normal distribution and used a paired sample *t*-test to compare c-HOAs at postoperative stage to c-HOAs at preoperative stage. All statistical analysis was done using the statistical software package STATA (version 12.1, STATA Corporation, College Station, TX, USA). Plots were generated using ggplot2 of the R-platform. *p*-values below 0.05 were considered strong evidence for an effect.

## Results

### Demographics

A total of 53 patients with a mean age 74.1 years (SD 8) were enrolled. Thirty-three patients were female (62.3%). Surgery was performed on 69 eyes: Group A: 27 eyes (21 patients); group B: 25 eyes (15 patients); group C: 21 eyes (17 patients). Complete datasets were available for all eyes.

### Refractive outcomes and visual acuity

Baseline analysis showed similar refractive values across all groups except for astigmatism. Astigmatism was highest in group C but comparable in group A and B (Table 1). Mean BCVA was low in all groups without important differences between the three groups (*p* = 0.49).

**Table 1.** Baseline and postoperative characteristics stratified by groups.

	Preoperative			Postoperative		
	Group A (Phaco)	Group B (FLACS)	Group C (FSAK)	Group A (Phaco)	Group B (FLACS)	Group C (FSAK)
	*N* = 27	*N* = 25	*N* = 21	*N* = 27	*N* = 25	*N* = 21
	Mean (SD)	Mean (SD)	Mean (SD)	Mean (SD)	Mean (SD)	Mean (SD)
Spherical equivalent (D)	−0.95 (4.51)	−0.69 (3.14)	−0.64 (4.62)	−0.54 (0.96)	−0.14 (0.38)	−0.32 (0.88)
Visual acuity (logMAR)	0.33 (0.15)	0.34 (0.16)	0.43 (0.31)	0.02 (0.06)	0.02 (0.06)	0.10 (0.14)
Axis length (mm)	23.98 (1.61)	23.48 (1.39)	23.76 (1.60)	24.86 (4.69)	23.38 (1.46)	23.50 (1.73)
Flat K (D)	42.89 (1.59)	42.73 (1.41)	42.68 (1.50)	42.77 (1.77)	40.72 (1.46)	42.80 (1.67)
Steep K (D)	43.52 (1.61)	43.23 (1.38)	43.82 (1.70)	43.7 (1.70)	43.29 (1.42)	43.72 (1.78)
Cylinder (D)	−0.67 (0.50)	−0.49 (0.45)	−1.13 (0.60)	−1.00 (0.77)	−0.70 (0.57)	−0.89 (0.58)
Axis (°)	70.61 (66.95)	82.57 (63.30)	87.10 (58.00)	101.64 (64.87)	77.60 (47.72)	90.20 (47.91)
Total cHOA mean in µm (SD)	0.277 (0.110)	0.269 (0.104)	0.345 (0.218)	0.353 (0.125)	0.335 (0.151)	0.460 (0.201)

cHOA: corneal higher-order aberrations; FLACS: femtosecond laser-assisted cataract surgery; FSAK: femtosecond laser-assisted cataract surgery with astigmatic keratotomy; K: keratometry; logMAR: logarithm of the minimum angle of resolution; µm: micrometer; SD: standard deviation.

At post-surgery, mean BCVA increased significantly in all groups (all groups: *p* ⩽ 0.001) without evidence for a difference between the three groups (*p* = 0.068). The mean postoperative spherical equivalent showed no differences between all groups (*p* = 0.586).

At post-surgery, astigmatism increased slightly in group A and B but decreased in group C (Table 1).

### Corneal higher-order aberration

**Figure 1.**
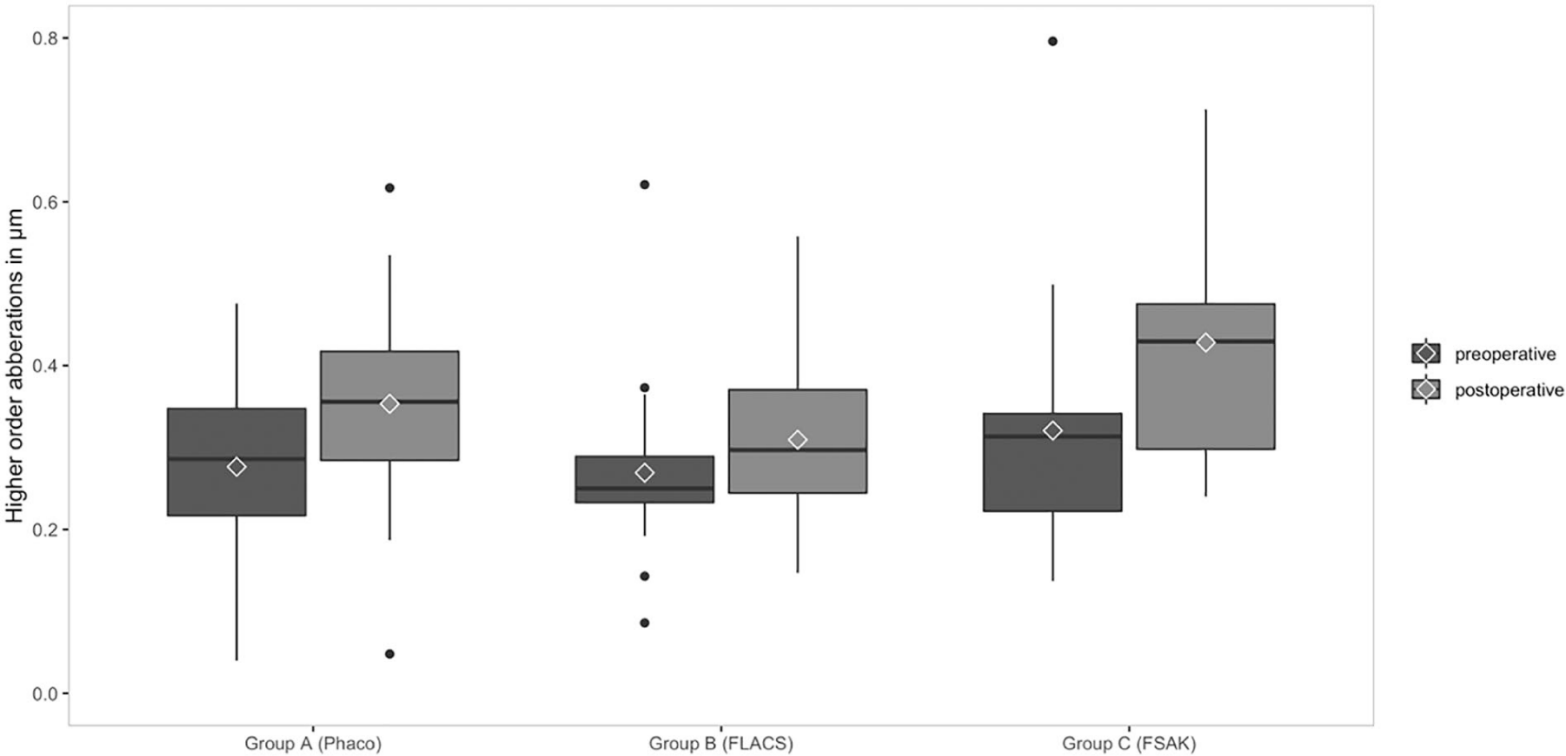
Comparison of corneal higher-order aberration in group A: conventional manual phacoemulsification (Phaco), group B: femtosecond laser-assisted cataract surgery (FLACS), and group C: femtosecond laser-assisted cataract surgery with astigmatic keratotomy (FSAK).

Comparing total c-HOAs between the groups showed no evidence for a difference prior to surgery (*F*(2,66) = 2.2, *p* = 0.128), but some evidence for a difference after surgery (*F*(2,65) = 3.87, *p* = 0.025). After surgery, total c-HOA increased in all groups (group A: *p* = 0.003; group B: *p* = 0.019; group C: *p* = 0.067), with the greatest c-HOAs increase seen in group C (FSAK) (Figure 1). The amount of postoperative variation in c-HOA was highest in group C (FSAK.

When examining different types of c-HOAs, we found no evidence for an increase of c-HOA in group A (Phaco) (Table 2). In group B (FLACS), some evidence suggested an increase in Trefoil, whereas in group C (FSAK), some weak evidence was found for an increase in spherical aberration (Table 2).

**Table 2.** Mean difference in corneal higher-order aberration pre- and post-surgery.

	Mean difference	95% CI	*p*-value
Group A (Phaco)			
Coma	−0.05	−0.13 to 0.37	0.265
Spherical	0.01	−0.17 to 0.04	0.371
Trefoil	−0.02	−0.11 to 0.06	0.660
Group B (FLACS)			
Coma	−0.03	−0.08 to 0.16	0.261
Spherical	−0.01	−0.04 to 0.02	0.681
Trefoil	−0.07	−0.13 to 0.14	0.015
Group C (FSAK)			
Coma	0.03	−0.08 to 0.13	0.621
Spherical	0.02	−0.27 to 0.06	0.041
Trefoil	−0.05	−0.17 to 0.05	0.322

## Discussion

Several studies have shown potential advantages of the FLACS when compared to manual Phaco.^2–5^ One example is the reduction of phacoemulsification energy and effective phacoemulsification time (EPT) in comparison to conventional cataract surgery.^15–21^ Furthermore, FLACS is able to create a more circular and precise capsulorrhexis, which can facilitate phacoemulsification, IOL implantation, and provide more accurate refractive outcomes after surgery.^22^ Other studies report advantages in terms of endothelial cell loss, intraocular lens position, and corneal swelling.^23–25^

However, some studies investigated the differences between FLACS and conventional manual Phaco without demonstrating a superiority of either technique such as achieving better and faster visual rehabilitation and refractive outcomes.^26–30^

The aim of refractive cataract surgeons is to achieve good results not only in lower-order aberrations (sphere and cylinder), but also in the outcome of c-HOAs. A reduction of spherical aberration can be achieved with the implantation of AIOL, which can lead to higher contrast sensitivity and a better retinal image quality.^9–13^

Denoyer et al.^31^ demonstrated that the corneal incision changes the morphology and is able to induce aberrations. The study of Marcos et al.^32^ postulated that with larger incision size, a higher value of c-HOA can be seen. In contrast, in our previous study Von Sonnleithner et al.^14^ showed in a study comparing three different incision sizes, 1.4 mm, 1.8 mm, and 2.2 mm, that the 1.8 mm incision induces less corneal HOA. In our current study, the incision size was 2.2 mm in all three groups.

Some studies reported that the difference of BCVA and uncorrected distance visual acuity (UDVA) between FLACS and manual Phaco were minimal to non-existent.^33,34^ In contrast, another study reported that FLACS achieved better visual outcome at 6 months of follow-up.^27^ In addition, the incidence of relevant complications, such as intraoperative anterior capsule tear, postoperative macular edema, and elevated intraocular pressure did not increase.^27^ However, in order to achieve best visual outcome, the c-HOAs must also be taken into account.

The main purpose of this trial was to investigate the effect of the cataract surgery technique (manual Phaco vs FLACS vs FSAK) on c-HOAs. Our results show that manual Phaco and FLACS induce c-HOAs, to a similar degree. We were able to demonstrate that in FSAK the increase of c-HOA is higher compared to manual Phaco and FLACS.

FSAK is useful in the reduction of corneal astigmatism and is comparable to toric intraocular lens implantation in eyes with low to moderate astigmatism.^35^ As we know, HOAs are part of refractive errors, but they are not correctable with sphere and cylinder corrections. They can impair the quality of the retinal image^36,37^ and lead to symptoms such as difficulty with night vision, glare, halos, blurring, starburst patterns, and diplopia. Thus, c-HOAs have an impact on visual performance and on contrast sensitivity.^38–40^

One of the advantages of astigmatic keratotomies over toric IOLs is that there is no risk of unwanted IOL-rotation. Rotation leads to less correction of astigmatism and can induce a hyperopic shift.^41^ The correcting effect is even eliminated when rotating 30°.^42^

Chan et al.^43^ investigated the stability of corneal astigmatism and c-HOAs after FSAK in 50 eyes of 50 patients. The mean of preoperative corneal astigmatism was 1.35 +/− 0.48 D, which could be reduced to 0.67 ± 0.54 D after 2 months and 0.74 ± 0.53 D after 2 years postoperatively. But the reduction in astigmatism was accompanied by an increase in c-HOAs.^43^ In this study, the surgeons used a single FSAK opposite to the main corneal incision. In our clinical setting, the main corneal incision (in groups B and C) was always placed in superior position. In dependence of the steep meridian, we placed one or two astigmatic keratotomies. Due to the limited number of eyes, we did not analyze the differences between these two possibilities.

Lee et al.^44^ compared conventional Phaco with FLACS in his study. In all eyes, a multifocal IOL was implanted. In addition, patients in the FLACS group with corneal astigmatism greater than 0.75 D also had arcuate keratotomy. Corneal higher-order aberrations were significantly higher in the FLACS group. In contrast to our study, it is important to note that in the FLACS group no difference was made between patients with and patients without arcuate keratotomy. In addition, satisfaction scores were significantly higher in the FLACS group.^44^

Another advantage of FLACS is that without further effort astigmatism (with astigmatic keratotomy) can be reduced. In comparison to toric IOLs, no preoperative calculation and order of the appropriate toric IOL is necessary.

We did not fully investigate the effect of corneal HOAs on the actual visual acuity of the patients. The aim of this study, however, was not to detect difference in visual acuity or astigmatism as this would have meant a very large sample size, but to explore a possible impact of the three different techniques on c-HOA.

Another source of bias could come from the different injectors and cartridges required for the respective IOLs used in this study. It is possible, that the different system had an effect on c-HOA and hence confounded our findings. Baseline analysis, however, showed that the different IOLs used in this study were randomly distributed across all groups. This means that a possible effect of the IOL on c-HOA, would have affected our findings to a similar degree in all three groups and hence should not have compromised the comparison.

In conclusion, all three groups showed an increase in c-HOAs, while FSAK demonstrated the highest increase. In patients with high values of c-HOA and low to moderate regular astigmatism preoperatively, a further increase of c-HOAs with FSAK should be considered. In such cases, implantation of toric IOLs may help to avoid an increase in postoperative c-HOAs.
